# Associative plasticity in supplementary motor area - motor cortex pathways in Tourette syndrome

**DOI:** 10.1038/s41598-018-30504-8

**Published:** 2018-08-10

**Authors:** Jennifer Tübing, Bettina Gigla, Valerie Cathérine Brandt, Julius Verrel, Anne Weissbach, Christian Beste, Alexander Münchau, Tobias Bäumer

**Affiliations:** 1 Institute of Neurogenetics, Department of Pediatric and Adult Movement Disorders and Neuropsychiatry, University of Lübeck, 23562 Lübeck, Germany; 2 Department of Neurology, University Medical Hospital of Schleswig-Holstein, 23538 Lübeck, Germany; 3 Centre for Innovation in Mental Health, Department of Psychology, University of Southampton, SO17 1BJ Southampton, England; 4 Cognitive Neurophysiology, Department of Child and Adolescent Psychiatry, Faculty of Medicine of the TU Dresden, 01307 Dresden, Germany

## Abstract

The important role of the supplementary motor area (SMA) in the generation of tics and urges in Gilles de la Tourette syndrome (GTS) is underscored by an increased SMA-motor cortex (M1) connectivity. However, whether plasticity is also altered in SMA-M1 pathways is unclear. We explored whether SMA-M1 plasticity is altered in patients with Tourette syndrome. 15 patients with GTS (mean age of 33.4 years, SD = 9.9) and 19 age and sex matched healthy controls were investigated with a paired association stimulation (PAS) protocol using three transcranial magnetic stimulation (TMS) coils stimulating both M1 and the SMA. Standard clinical measures for GTS symptoms were collected. There was a significant PAS effect showing that MEP amplitudes measured in blocks during and after PAS were significantly higher compared to those in the first block. However, the degree of PAS was not differentially modulated between patients and controls as shown by a Bayesian data analysis. PAS effects in GTS correlated positively with the YGTSS motor tic severity. Plasticity previously reported to be altered in sensorimotor pathways in GTS is normal in SMA-M1 projections suggesting that the dysfunction of the SMA in GTS is not primarily related to altered plasticity in SMA-M1 connections.

## Introduction

Gilles de la Tourette syndrome (GTS) is a multifaceted neuropsychiatric disorder characterized by multiple motor and vocal tics lasting for more than a year and starting before the age of 18^1^. Tics are repetitive, patterned movements resembling voluntary movements but appearing misplaced in context and time^2,3^. They are often preceded by an urge to move that has thus been considered as a core event driving tics^4,5^. It is generally agreed that abnormalities in cortico-striato-thalamo-cortical (CSTC) loops with an imbalance of inhibitory and excitatory pathways play a key role in the pathogenesis of GTS^6^. In particular, the supplementary motor area (SMA) appears to be a crucial relay. For instance, it has been demonstrated that the SMA is activated in the seconds preceding tics^7–9^. Increased activation of the SMA has also been linked to sensory urges (10–12). Several, though imperfectly controlled, studies provided preliminary evidence that inhibitory (1 Hz) repetitive TMS delivered to the SMA decreases tic frequency^10–14^ suggesting that over-activity of the SMA is related to tic frequency. In addition, both fMRI and MEG have shown increased functional connectivity between SMA and the primary motor cortex (M1) in GTS patients^9,15^. It therefore seems that the neural dynamics and the connectivity between SMA and M1 circuits are central for the understanding of tics and GTS.

In this respect, it is important to consider that (functional) connectivity aspects are closely related to plasticity mechanisms of neuronal circuits^16–18^. However, the question whether plasticity in SMA-M1 circuits is also altered in GTS has not been addressed. This is a relevant issue because motor learning, which is related to plasticity^19^, has been shown to be altered in GTS^20^. Importantly, some data suggest that impaired learning of fine motor skills is related to future tic severity^7^ and increased habit formation tendencies have also been related to tic severity^21–23^.

Given the prominent role of the SMA in the generation of tics and urges, increased SMA-M1 connectivity, altered plasticity within M1 and sensorimotor pathways in GTS^20,24,25^ and tic-related abnormalities of motor learning, we here investigated plasticity in SMA-M1 circuits in GTS patients. Long-term potentiation (LTP) and long-term depression like plasticity can experimentally be induced in M1 in humans using repetitive TMS protocols including theta burst stimulation (TBS), high frequency stimulation and paired associative stimulation (PAS)^26–29^. Given some evidence that PAS induces synaptic plasticity more effectively than TBS^30^ it appears to be the most suitable methods to induce plasticity, particularly against the background that variability of responses in plasticity protocols is often high^31–33^. We examined SMA-M1 plasticity using an MR-navigated SMA-M1 PAS protocol that has previously been established by Arai *et al*. in healthy controls through pairing of TMS pulses applied to the SMA with TMS pulses given over M1^34^. These authors showed that LTP-like effects can be induced in SMA-M1 circuits in a timing-dependent, directional and region-specific fashion^34^. The main aim of the present study was to explore whether known abnormalities of functional connectivity in SMA-M1 projections in GTS^9,15^ are associated with altered plasticity in these circuits as determined with this SMA-M1 plasticity protocol.

## Results

### Clinical Data

At the time of the study, all patients reported currently having motor tics and seven reported currently having phonic tics. Mean (SD) DCI score was 42.1 (8), mean YGTSS total tic severity was 13.5 (3.7), mean YGTSS motor tic severity was 10.8 (3.6), mean YGTSS phonic tic severity was 2.7 (3.1), mean PUTS score was 18.7 (4) and mean MRVS was 8.8 (1.6) (see Table 1).

**Table1:** Clinical data and measures of Tourette syndrome severity.

Patient	Age	Sex	Medication	DCI	PUTS	MRVS	YGTSS	YGTSS	YGTSS
							Motor Severity Score	Phonic Severity Score	Total Score
P02	33	m	no	33	21	11	9	8	17
P03	28	f	no	57	18	9	10	3	13
P05	29	m	Citalopram	32	22	5	9	0	9
P06	45	m	L-Thyroxin	43	16	9	14	3	17
P07	20	m	no	49	20	8	14	0	14
P08	26	m	no	38	22	10	15	0	15
P09	38	m	no	42	15	8	12	0	12
P10	38	m	no	46	12	8	5	0	5
P11	26	m	no	34	15	9	9	7	16
P12	56	m	no	n.a.	n.a.	8	6	5	11
P13	29	m	no	n.a.	26	10	17	0	17
P15	25	m	no	47	19	11	10	6	16
Mean	32.8			42.1	18.7	8.8	10.8	2.7	13.5

DCI = Diagnostic Confidence Index; MRVS = total score of Modified Rush Videotape Rating Scale; N.a. = not available, m = male, f = female; PUTS = Premonitory Urge for Tics Scale; YGTSS = Yale Global Tic Severity Scale Score.

### Transcranial magnetic stimulation

**Figure 1:**
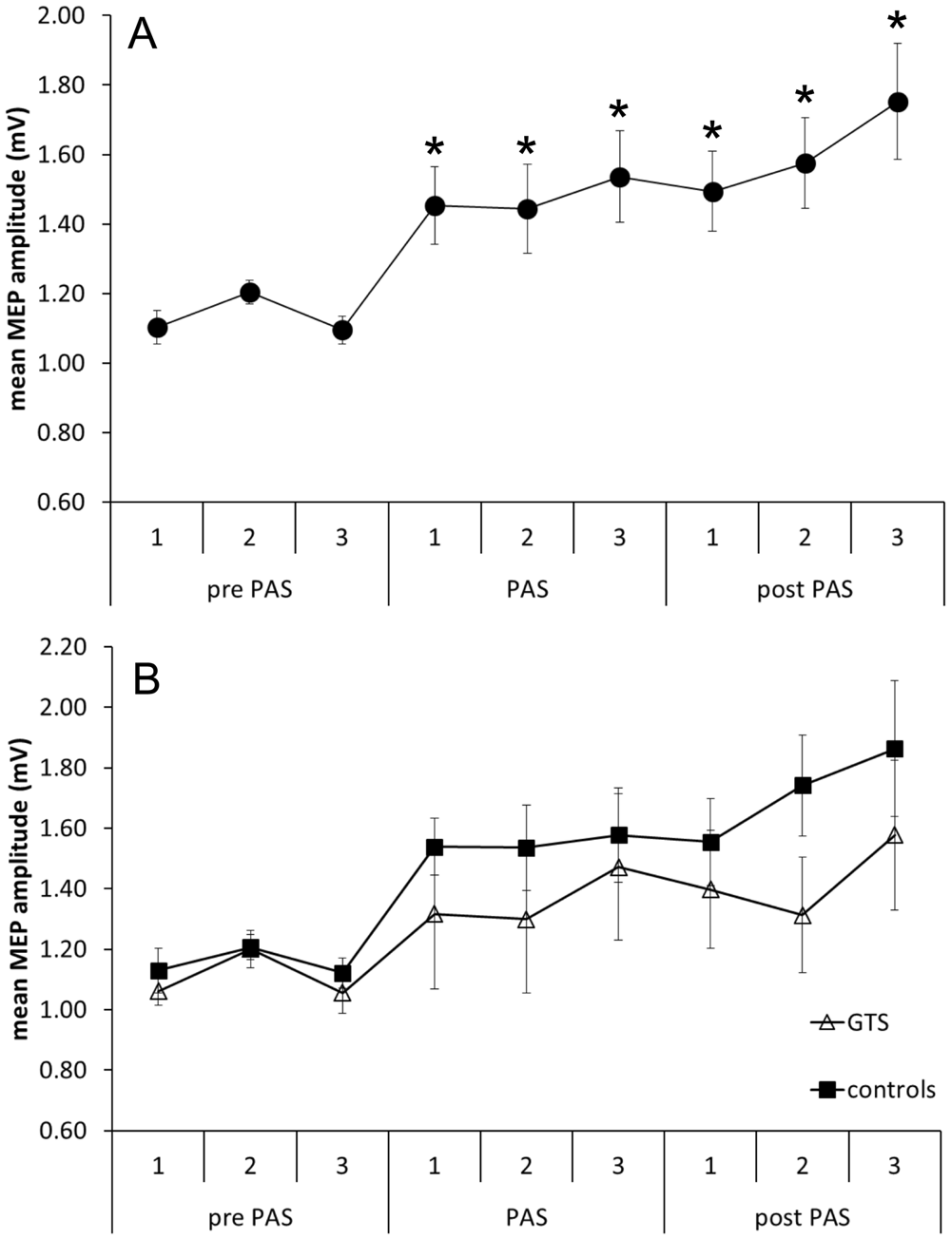
Effects of SMA stimulation in GTS patients and healthy controls. MEP amplitudes before (pre SMA), during (PAS) and after (post SMA) associative stimulation of SMA paired with left M1. (**A**) PAS effects of both groups combined. Asterisks indicate a significant effect compared to the first bock. (**B**) MEP amplitudes of healthy controls compared to those of Gilles de la Tourette syndrome (GTS) patients.

The stimulation protocol consisted of a total of 450 trials, grouped into three parts (pre PAS, PAS, post PAS) with three blocks of 50 trials each. ANOVA with MEP amplitude as dependent variable revealed no main effect for GROUP (F(1, 29) = 1.13; p = 0.297) but a significant effect of CONDITION (pre PAS, PAS, post PAS; F(2, 58) = 9.92; p < 0.001), no effect of BLOCK (F(2, 58) = 1.92; p = 0.16), no interaction of CONDITION × BLOCK (F(4, 116) = 2.5; p = 0.71), no interaction of the factor GROUP with CONDITION ((F(2, 58) = 0.69; p = 0.5) or BLOCK (F(2, 58) = 0.27; p = 0.76), and no three-way interaction (F(4, 116) = 0.8; p = 0.53). Post hoc analyses of the PAS effect of all 9 blocks revealed that MEP amplitudes in blocks measured during PAS and after PAS were significantly larger compared to those in the first block (p < 0.05; Bonferroni-Holm corrected) (Fig. 1a).

The absence of a significant GROUP × CONDITION interaction was further analyzed using Bayesian statistics^35^. The approach proposed by Masson *et al*. allows estimating the relative evidence for different statistical models from sums-of-squares data used in the ANOVA^36^. For the GROUP × CONDITION interaction (SS_effect = 0.659, SS_Error = 27.487, Bayes Factor of 21.48), the analysis revealed a probability of the null hypothesis of p(H0|D) = 0.955, corresponding to a Bayes Factor of 21.48 in favor of the null hypothesis. This indicates that given the data, the null hypothesis (no GROUP × CONDITION interaction) is about 20 times more likely than the alternative hypothesis (presence of the interaction), thus providing strong evidence for the null hypothesis according to the criteria by Raftery^37^; i.e. that PAS had no differential effect in the two groups.

### Relation to clinical measures

**Figure 2:**
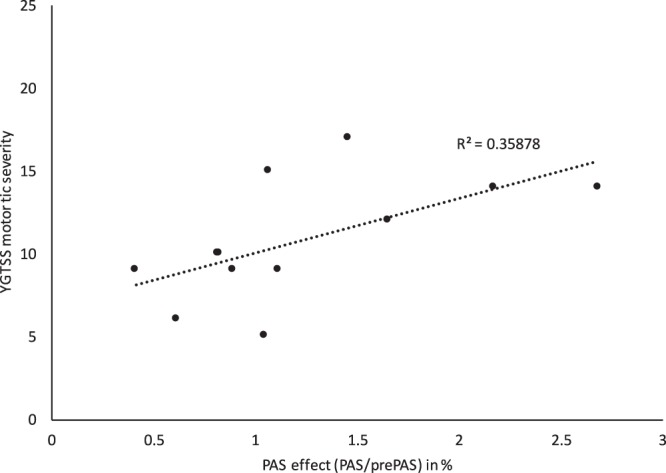
Distribution plot for the correlation between PAS effects and the YGTSS motor tic severity scores (p = 0.048, r = 0.58).

The YGTSS Motor Tic severity score (p = 0.031, r = 0.62) (Fig. 2) correlated positively with MEP changes from the condition pre PAS to PAS in GTS patients suggesting that tic severity was associated with the degree of the PAS effect. There were no other significant correlations of clinical scores with PAS effects (p > 0.05).

## Discussion

The main findings of the present study are that (i) PAS in SMA-M1 circuits did not differ between GTS patients and healthy controls and that (ii) the amount of LTP-like effects correlated with current motor tic severity in patients. Thus, although abnormalities of functional connectivity have been demonstrated in SMA-M1 projections in GTS^9,15^ there are no alterations in the plasticity of these connections, at least when tested with a PAS protocol as used here.

This is an important finding, since previous studies employing intermittent and continuous theta burst TMS and inhibitory high-frequency electrical stimulation of the supraorbital nerve reported that plasticity is reduced in GTS patients^25,38^. Likewise, using a PAS protocol where peripheral electrical stimulation of the median nerve was coupled with TMS over M1, Brandt *et al*. showed that on average there was no typical LTP-like effect in response to PAS in these patients^20^. These studies, although using different methodology, suggest reduced plasticity in brainstem circuits^25,38^ and also in sensorimotor pathways^20^. However, in another PAS study, using the same protocol as Brandt *et al*.^20^, Martin-Rodriguez *et al*.^24^ found that LTP-like effects were stronger in GTS patients compared to healthy controls.

Besides the high variability of stimulation effects in non-invasive brain stimulation studies mentioned above^31–33^, the diversity of findings may be explained by variations in tic severity in the different studies. Tic severity of patients in the study by Martin-Rodriguez *et al*. was considerably higher (mean YGTSS score of 43.9) compared to the study by Brandt *et al*. (mean YGTSS score of 15.7)^20,24^. Importantly, in both studies LTP-like effects were associated with tic severity. Although clinical severity scores did not correlate with plasticity measures in the studies by Suppa *et al*.^25,38^, clinical scores were rather low in these studies (YGTSS score between 23.5 and 18.5) where plasticity was shown to be reduced. Although different protocols were used in studies addressing plasticity in GTS, it seems that there is reduced plasticity in patients with mild to moderate symptoms and abnormally increased plasticity in those with more severe symptoms. GTS patients included in the present study were also only mildly affected with a mean YGTSS scores of 13.5. This notwithstanding, their response in the SMA-M1 plasticity protocol was not attenuated, but in fact did not differ from healthy controls. This indicates that there is no global reduction of plasticity in neural networks in GTS, but apparently altered plasticity predominantly in sensorimotor and brainstem circuits in GTS.

Interestingly, structural abnormalities are also only found in some brain networks in GTS including the pre-frontal and cingulate cortex, the basal ganglia, the sensorimotor cortex and the corpus callosum^39–45^. The largest currently available data set showed grey matter increases in the midbrain and thalamus and lower white matter volume in orbitofrontal regions^46^. Other data particularly emphasize abnormal, i.e. predominantly increased connectivity, in cortico-striato-pallido-thalamo-cortical networks^42^. Less is known about small range cortico-cortical networks including, for instance, SMA-M1 connections. It is therefore possible, that these connections mediating SMA-M1 plasticity are largely intact in GTS. Even if there were structural abnormalities in SMA-M1 pathways in GTS, these might not necessarily be associated with altered plasticity. In any case, well-known abnormalities in the SMA related to the generation of tics and urges in GTS^7–9^ are apparently not related to altered plasticity processes in this area. Future studies shall therefore examine the interrelation of structural alterations and neural plasticity in GTS to provide structural neural plasticity maps to examine whether this may also explain the severity of tics in GTS. In that regard, it is interesting that in both classical PAS testing plasticity in sensorimotor pathways^20,24^ and SMA-M1 PAS, testing SMA-M1 connections (present study), LTP-like responses were correlated with measures of tic severity. The results could be interpreted such that LTP-like effects are dependent on tics, i.e. more severe tics lead to stronger LTP. Yet, if so, GTS patients should generally have stronger LTP-like effects than healthy controls. This is not the case. Taken together, it is likely that tics per se do not affect LTP- or LTD like plasticity but rather other as yet to be determined factors influencing both tics and LTP-like plasticity. One factor could be compensatory mechanisms during brain maturation to control for tics resulting in stronger LTD like plasticity in patients with lower tic scores. Thus, more effective tic inhibition leading to reductions in tic severity might be related to a tendency of the sensorimotor system for LTD like plasticity as a response to excitability altering influences including experimental interventions. Such a mechanism would be in line with MRI findings showing different short and large range connectivity patters in GTS compared to healthy controls^47,48^.

In addition, it is possible that motor learning, which is closely related to plasticity^19^, also plays an important role. In this respect, it is interesting to note that tics bear resemblance to habits, i.e. overlearned actions and action sequences^49^. In fact, recent studies have shown an increased habit formation tendency in GTS patients correlating with structural abnormalities, i.e., increased connectivity between the basal ganglia and motor cortical regions^50^. In addition, there is some experimental evidence that tics resemble over-learned behavior^51^. If motor learning capacity is in fact the underlying driving force, of both tics and LTP-like responses in PAS protocols then it is perhaps not surprising that differences between healthy controls and GTS patients become more apparent in protocols where basal ganglia thalamo-cortical loops are implicated than in protocols primarily testing SMA-M1 connections as in the present study.

Taken together, our data suggest that the SMA is not primarily implicated in the (learning associated) formation of tics or the propensity to develop tics, which is probably predominantly determined by the basal ganglia^52^. This does not generally question the crucial role of the SMA in the pathophysiology of tics. The SMA might be particularly relevant with respect to tic occurrence and the inner structure of tics, because the SMA is physiologically engaged in the preparation and temporal organization of self-initiated movements^53–57^.

## Conclusions

In contrast to previous studies documenting altered plasticity in sensorimotor pathways and brainstem circuits in GTS the present study shows that plasticity in SMA-M1 pathways is normal in these patients. This indicates that well-established dysfunctions of the SMA in GTS are not primarily related to altered plasticity processes associated with the propensity to tic.

## Materials and Methods

### Subjects

Fifteen patients (mean age of 33.4 years, SD = 9.9, age range = 20–56 years; one female) with a diagnosis of GTS according to DSM-V criteria^58^ were recruited from the University Medical Center in Lübeck, Germany. Due to the fact that the experimental paradigm requires subjects to sit relaxed over an extended period of time without moving the head, we only recruited patients with mild or moderate tics. Three of these patients were excluded from the analyses because of severe motor tics during TMS measurements obviating correct positioning of the TMS coils. All patients had uncomplicated GTS without significant clinically relevant comorbidities. Patients fulfilling criteria for OCD, ADHD or other neurological or psychiatric comorbidities were excluded from the study.

Nineteen age- and sex matched healthy subjects were recruited as a control group (mean age 31.4 years, SD = 10.4, age range = 20–51 years; one female). They had no history of psychiatric disorders or neurological diseases.

All subjects were right-handed according to the Edinburgh Handedness Inventory^59^ except for two (one patient and one healthy control), who were left-handed. Written informed consent was obtained before participation. The experiments conformed to the Declaration of Helsinki and were approved by the local ethics committee of the University of Lübeck.

### Clinical Assessments

GTS symptom severity within a week before the study was assessed using the Yale Global Tic Severity Scale (YGTSS)^60^. To measure the lifetime likelihood of having GTS we used the Diagnostic Confidence Index (DCI)^61^. Standardized video recordings were performed and data were scored using the Modified Rush Videotape Rating Scale (MRVS)^62^. Two patients were taking medication at the time of the study. One patient had a history of a depressive episode in the past and was taking Citalopram, the other was taking L-Thyroxine because of hypothyroidism. Information about premonitory urges, assessed by the validated German version of the Premonitory Urge for Tics Scale (PUTS)^63^ was available for 12 patients. The PUTS was originally developed for children but has recently been validated also in adult GTS patients^64,65^. ADHD symptoms were rated on the ADHD self-rating scale^66,67^, while OCD symptoms were measured using the Obsessive-Compulsive Inventory (OCI)^68^. Clinical data and GTS scores are given in Table 1.

### Study design

Prior to the TMS experiments, all participants underwent an MRI scan to obtain a high resolution T1 weighted MRI. This was used for neuronavigated positioning of the TMS coils over the SMA using Brainsight (Roque Research Inc. Montreal; Canada). All participants completed a TMS safety screening. Thereafter, the SMA-M1 conditioning protocol was administered.

### Magnetic resonance imaging

Structural MRI of the whole brain was performed using a Siemens Magnetom Trio 3 T scanner equipped with a 32-channel head coil. High resolution T1-weighted anatomical images for each subject were obtained using a standard 3D MP-RAGE sequence (TR = 2300 ms; TE = 2.98 ms; TI = 1100 ms; flip angle 9°; 1 × 1 × 1 mm^3^ spatial resolution; 240 coronal slices, field of view 192 × 256 mm^2^).

### Transcranial magnetic stimulation

Subjects were seated in a comfortable armchair. A TMS coil and subject head holder (Brainsight TMS frame; Roque Research Inc. Montreal; Canada) were adjusted to a frame surrounding the subjects’ chair. The head holder fixed to the frame allowed for a comfortable sitting position with the subjects’ heads resting on the holder and neck muscles relaxing. The coil holder ensured an accurate positioning of the TMS coils onto the subjects’ heads. The arms of the subjects were supported by pillows, so that arm muscles were relaxed. During all recordings, subjects were requested to relax but stay awake and keep their eyes open throughout the experiment.

Surface electromyography (EMG) was recorded from resting right and left first dorsal interosseous (FDI) muscles using pairs of electrodes in a belly-tendon montage. The ground electrode was placed at the wrist. The EMG raw signal was amplified and filtered using a bandpass filter from 20 Hz to 1 kHz with a D360 amplifier (Digitimer Limited, Welwyn Garden City, UK). The signals were sampled at 5000 Hz, digitized using a laboratory interface (Micro1401, Cambridge Electronics Design (CED), Cambridge, UK) and stored on a personal computer for display and later off-line data analysis. EMG signals were continuously monitored acoustically with loudspeakers and visually by means of an oscilloscope.

Measurements were performed with three Magstim 200 magnetic stimulators, each of them connected with a figure-of-eight shaped coil with handles perpendicular to the coil windings (“Branding-Iron-Style”). Coils for stimulating M1 had an outer winding diameter of approximately 50 mm of each wing, those used for stimulation of SMA had an outer winding diameter of approximately 25 mm of each wing (Magstim Company, Whitland, Dyfed, UK). For M1 stimulation, the coils were placed tangentially to the scalp at a 45° angle away from the midline, approximately perpendicular to the line of the central sulcus inducing a posterior to anterior directed current in the brain. We determined the optimal position for activation of the FDI by moving the coil in 0.5 cm steps around the presumed primary motor hand area of both hemispheres. The sites where stimuli of slightly supra threshold intensity consistently produced the largest MEPs with the steepest negative slope in the corresponding first dorsal interosseous muscle (referred to as “motor hot spot”) were marked with a wax pen. TMS coils for M1 stimulation were fixed to the frame using coil holders and placed at the marked stimulation sites onto the subjects’ head.

**Figure 3:**
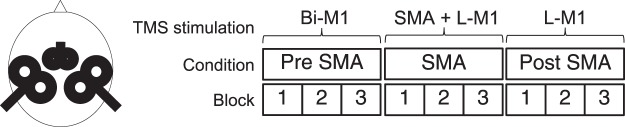
(**A**) Coil positioning in the experiment. (**B**) General time line of the experiment, always consisting of nine blocks of 50 trials each (Pre PAS, PAS, post PAS). The mean duration of each block of 50 trials was 5 min. followed by a short break of about 2 min to allow for cooling/changing of the stimulation coils. During PAS, TMS of SMA-proper was given 6 ms before left M1 stimulation. in Pre PAS blocks M1 excitability was determined at baseline, during PAS paired associative stimulation was applied to SMA and left M1. In the post PAS blocks lasting effects on M1 excitability after PAS were assessed. Stimulation procedure is detailed in Materials and Methods. Bi-M1 = bilateral M1 stimulation; L-M1 = left M1 stimulation; SMA = SMA-proper.

With coils fixed to the frame we determined the resting and active motor threshold (MT). Resting MT was defined as the minimum stimulus intensity that produced an MEP of more than 50 mV in 5 out of 10 consecutive trials. Active MT was defined as the lowest stimulus intensity at which MEPs were elicited in 5 out of 10 consecutive trials during a tonic contraction of the FDI muscle at about 10% of maximum force level and a criterion for the MEP of 100–150 µV peak-to-peak amplitude. The intensity of the test pulse was set at an intensity that, when it was given alone, would evoke an EMG response of approximately 1 mV peak-to-peak size in the left first dorsal interosseous muscle. The coil for the SMA stimulation was also held by a coil frame holder and placed on the subjects’ head according to the previously published Talairach coordinates of the SMA hand area (*x* = 0, *y* = −7, *z* = 52)^69^ on the individual MRI dataset using Brainsight TMS Navigation (Fig. 3). The orientation of the coil was rectangular to the midline and the resulting current flow in the brain was in a posterior to anterior direction. The intensity of the SMA pulse was set at 140% of AMT determined with the 25 mm coil over M1 in the above described way.

To test for potential current spread of the SMA conditioning pulse to M1 we tested SMA conditioning (20 trials) with 100% of the maximum stimulator output in 10 subjects and could not detect any MEP over the FDI. Thus, we feel confident that there is no direct spread to M1 during SMA conditioning when using a TMS intensity of 140% AMT, i.e. an intensity much lower than 100% of maximum stimulator output.

### Experimental design

The experiment comprised nine separate blocks of 50 trials each^34^(see Fig. 3). In blocks 1–3 (pre PAS), M1 was stimulated bilaterally with the above determined test pulse intensity (near-simultaneous with an interstimulus interval of 0.8 ms between M1 pulses). This served as pre-conditioning, which was previously shown to be necessary to induce subsequent SMA-M1 PAS^34^. In blocks 4–6, PAS was applied. SMA stimulation was given at an intensity of 140% of AMT. SMA and left M1 pulses were coupled such that SMA stimulation preceded left M1 stimulation by 6 ms. In blocks 7–9 (post PAS), MEPs following left M1 stimulation were determined. In each block, the inter trial interval was jittered at 5 s ± 25% to reduce anticipation of the next trial. The mean duration of each block of 50 trials was 5 min followed by a short break of about 2 min to allow for cooling/changing of the stimulation coils.

### Data analysis and statistics

MEP amplitudes were measured semi-automatically peak-to-peak for each frame using Signal software (customized script). Mean values were calculated for each participant by averaging the MEP amplitudes, excluding single trials that deviated more than 2.5 SDs from the mean.

Statistical analyses were performed using SPSS 22. We tested the PAS effect using a two-way ANOVA with the factors CONDITION (pre PAS, PAS, post PAS) and BLOCK (three blocks of 50 MEPs per CONDITION) with the in between subject factor GROUP (GTS patients and control). Greenhouse Geisser correction was used for non-sphericity. In addition, to classical null hypothesis testing (NHST) using mixed effects ANOVAs, we therefore also used Bayesian statistics^35,36,70,71^ to evaluate the relative strength of evidence for the null and alternative hypothesis.

The scores of the clinical tic and urge measures (YGTSS, DCI, MRVS, PUTS) were correlated with the magnitude of the PAS effect (mean MEP value PAS / pre PAS) using Spearman Rho rank correlations. Because 5 patients participating in our study had no vocal tics at the time of the experiments or the week before correlations of scores including vocal tics were omitted. Effects were considered statistically significant if p < 0.05. Post hoc test were corrected for multiple testing using Bonferroni – Holm correction^72^.
